# Public health impact and economic benefits of quadrivalent influenza vaccine in Latin America

**DOI:** 10.1080/21645515.2016.1256928

**Published:** 2017-01-24

**Authors:** Aurélien Jamotte, Emilie Clay, Bérengère Macabeo, Andrès Caicedo, Juan Guillermo Lopez, Lucia Bricks, Martín Romero Prada, Rubén Marrugo, Pamela Alfonso, Brechla Moreno Arévalo, Danilo Franco, Lourdes Garcia Diaz, Yadira Isaza de Molto

**Affiliations:** a Creativ-Ceutical, Paris, France; b Sanofi Pasteur, Lyon, France; c Sanofi Pasteur, Bogota, Colombia; d Sanofi Pasteur, Mexico, Mexico; e Sanofi Pasteur Latin America, Sao Paulo, Brazil; f Salutia Foundation, Bogota, Colombia; g The Gorgas Memorial Institute for Health Studies, Panama City, Panama; h Ministry of Health, Panama City, Panama

**Keywords:** influenza, vaccine, quadrivalent, Panama, Brazil, Colombia, Latin America, cost, benefit, public health

## Abstract

Annual trivalent influenza vaccines (TIV) containing 2 A strains and one B lineage have been recommended for the prevention of influenza in most of Latin American countries. However, the circulation of 2 B lineages (Victoria and Yamagata) and difficulties in predicting the predominating lineage have led to the development of quadrivalent influenza vaccines (QIV), including both B lineages. Thus, the objective was to estimate the public health impact and influenza-related costs if QIV would have been used instead of TIV in 3 Latin American countries. We used a static model over the seasons 2010–2014 in Brazil, 2007–2014 in Colombia and 2006–2014 in Panama, focusing on population groups targeted by local vaccination recommendations: young children, adults with risk factors and the elderly. In Brazil, between 2010 and 2014, using QIV instead of TIV would have avoided US$ 6,200 per 100,000 person-years in societal costs, based on 168 influenza cases, 89 consultations, 3.2 hospitalizations and 0.38 deaths per 100,000 person-years. In Colombia and Panama, these would have ranged from US$ 1,000 to 12,700 (based on 34 cases, 13–25 consultations, 0.6–8.9 hospitalizations and 0.04–1.74 deaths) and from US$ 3,000 to 33,700 (based on 113 cases, 55–82 consultations, 0.5–27.8 hospitalizations and 0.08–6.87 deaths) per 100,000 person-years, respectively. Overall, the broader protection offered by QIV would have reduced the influenza humanistic and economic burden in the 3 countries. Despite the lack of local data leading to several extrapolations, this study is the first to give quantitative estimates of the potential benefits of QIV in Latin America.

## Introduction

Influenza is an acute infectious respiratory disease caused in humans mainly by influenza viruses A and B. For young children, the elderly, or adults with risk factors such as people with severe chronic conditions, an infection can lead to severe complications of the underlying condition, pneumonia or even death.^1^ Worldwide, it is estimated that the annual influenza epidemic results in 3–5 million cases of severe illness and between 250,000–500,000 deaths.^2^ In Latin America, the annual incidence of influenza-like illness per 100,000 person-years was estimated to be 36,000, with between 4.7% and 15.4% influenza positive specimens depending on the influenza centers.^3^

Vaccination remains the most effective measure for preventing influenza and its complications.^4,5^ As of 2014, immunization against seasonal influenza was recommended in the public health policies of 40 out of 45 countries and territories in the Americas.^6^ In most countries, the standard vaccination procedure consists of the annual administration of trivalent influenza vaccine (TIV) containing 3 influenza strains: one A/H1N1 strain, one A/H3N2 strain and one influenza B strain (either from the Victoria or Yamagata lineages), which is intended to provide protection against influenza viruses expected to circulate in the upcoming influenza season. The World Health Organization (WHO) annually issues recommendations about the strains to be included in the TIV vaccine in the next season in the northern and southern hemispheres based on the reports provided by influenza surveillance networks worldwide. However, over the past years, 2 distinct lineages of influenza B (Yamagata and Victoria) have been co-circulating worldwide with one lineage dominating the other in many of the seasons.^7,8^ But, predicting which lineage will predominate in the next season has been revealed to be challenging, with frequent mismatches occurring between the lineage included in the TIV and the circulating lineage.^9^ For instance during the 2013 season in Brazil, the Yamagata lineage which was included in the TIV showed up as a mismatch since 89% of characterized influenza B viruses were from the B/Victoria lineage.^10^

Quadrivalent influenza vaccines (QIV), which include both B lineages, were designed to meet the challenge of the evolution in influenza epidemiology and to provide a direct additional benefit by guaranteeing a similar level of protection to TIV, whenever the circulating influenza B virus would not match the lineage included in TIV, either because the lineage prediction was incorrect or because both lineages co-circulated to a significant degree.

The public health and economic impacts of the administration of QIV instead of TIV in the US have been recently estimated by Reed et al.^11^ and Lee et al.^12^ through a hypothetical scenario where QIV would have replaced TIV over the period 1999–2008. The objective of this study was to build upon these 2 studies to estimate the additional benefit of using QIV rather than TIV on influenza-related health outcomes and associated costs in 3 countries of Latin America, a region with specific characteristics in terms of influenza circulation, healthcare systems and vaccination policies. More specifically, we estimated the additional impact of QIV in 3 countries: Brazil over 2010–2013, Colombia over 2007–2014 and Panama over 2006–2014, with season 2009 being excluded from the analysis due the H1N1 pandemic, which would have biased the results.

## Materials and methods

### Model description

**Figure 1.**
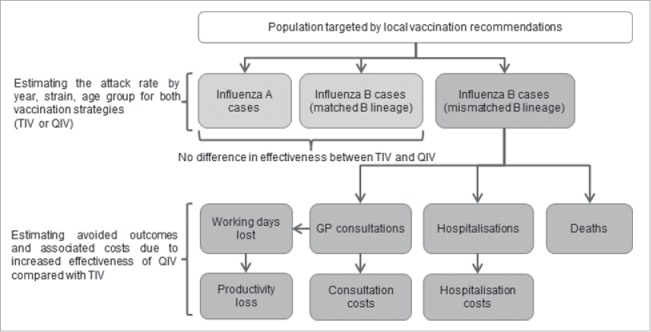
Model structure, GP: General Practitioner; QIV: Quadrivalent influenza vaccine; TIV: Trivalent influenza vaccine.

We developed an age-stratified static model which allowed the comparison of 2 different vaccination strategies: one, the actual situation where TIV was administered over the period of analysis for each country, and a second strategy where QIV would have replaced TIV. The numbers of influenza cases, General Practitioner (GP) consultations and associated work absenteeism, hospitalizations and deaths due to influenza, as well as their associated costs were estimated for each season and for both vaccination strategies in the 3 countries. A schematic representation of the model is available in Fig. 1.

Mathematically, the expected influenza attack rate attributable to a specific virus strain or lineage j (j=A, B/Yamagata, B/Victoria) in a population partially vaccinated with a vaccine i (i=QIV, TIV) for a given year was computed using the following formula: ${\text{AR}}_{\text{i},\text{j}}={\text{AR}}_{\text{no vac}}.{\text{p}}_{\text{j}}.\left(1-\text{VC}.{\text{VE}}_{\text{i}/\text{j}}\right)$, where ${\text{AR}}_{\text{no vac}}$ denotes the influenza attack rate expected without vaccination, ${\text{p}}_{\text{j}}\;$the proportion of strain j among all influenza strains, VC the vaccine coverage rate and ${\text{VE}}_{\text{i}/\text{j}}$ the effectiveness of vaccine i against strain j. The other influenza-related outcomes were derived proportionally from the age-specific numbers of influenza infections avoided.

The influenza-related costs avoided from the third-party payer (TPP) perspective (defined as the medical costs of GP consultations and hospitalizations) and the societal perspective (defined as the sum of medical costs supported by the TPP, including GP consultations and hospitalizations, and the loss of productivity due to work absenteeism associated to influenza cases requiring at least an outpatient consultation) were estimated by applying corresponding unit costs to the number of avoided events. Costs were computed in 2014 US dollars (US$) and the local currency of each country: Brazilian reals (BRL), Panamanian balboas (PAB) and Colombian pesos (COP) according to the 2014 average exchange rates: US$ 1 = BRL 2.35, US$ 1 = COL 2,002 and US$ 1 = PAB 1.13.

The analysis included different time horizons for each of the 3 countries, reflecting the availability of local influenza circulation data and history of influenza vaccination programs. The Brazilian analysis was performed for 2010–2014, which covered the period with the most robust data on influenza circulation. The analysis period for Colombia and Panama covered the period starting from the countrywide introduction of TIV in public vaccination campaigns (2006 for Panama and 2007 for Colombia), to 2014, the most recent year from which data on influenza circulation was available. For both countries, season 2009 was excluded from the scope due to the H1N1 pandemic, which rendered the year atypical and would have biased the estimated season-specific influenza attack rates.

Furthermore, in absence of local data to estimate the influenza burden in terms of GP consultations, hospitalizations and deaths in Brazil, we considered non local, US data that were estimated with robust methods, minimizing the reporting bias and the effects of miscoding that arise in most databases when estimating outcome rates due to influenza. The analysis for Colombia and Panama was performed on 2 different sets of inputs: one set using data from local databases and one set using more robust Colombian data extrapolated with US data. The two scenarios provide a range for the impact of QIV in which the true estimate probably lies.

## Data inputs

### Population

Some population groups are more likely to develop complications and even die as a result of their infection than others. Thus, the populations of analysis in the 3 Latin American countries corresponded to the population groups with increased risks of developing influenza complications, as defined in the most recent vaccination recommendations in the countries' Expanded Programs on Immunization (EPIs). In Brazil and Panama, vaccination recommendations included young children from 6 to 59 months, people aged 60 y and older, and people with risk factors;^14,15^ while Colombian authorities recommended vaccination of younger children (from 6 to 23 months) in addition to people aged 60 y and older, and people with risk factors.^16^ People with risk factors were defined as pregnant women and people with specific comorbidities, namely chronic respiratory diseases (asthma, chronic obstructive pulmonary disorder), cardiovascular diseases, metabolic diseases such as diabetes, chronic renal diseases, and people with immunodeficiency (based on the 2005 WHO position paper^17^). Local recommendations also included other population groups such as healthcare or community workers.

To reflect local vaccination recommendations and heterogeneity in the influenza burden, the model was stratified into 4 population groups: young children (6–59 months for Brazil and Panama, 6–23 months for Colombia), adults aged from 18 to 49 y with risk factors, adults aged from 50 to 59 y with risk factors and the elderly (60 y and older). Children with risk factors (5–17 y for Brazil and Panama, 2–17 y for Colombia), were not included in the scope of the study due to a lack of data, while other population groups for which vaccination was also recommended such as healthcare workers have not been included in the scope of the study as vaccination of these groups is primarily intended to prevent the disease from spreading in the population at risk, which was difficult to take into account in a static model.

For each country, the proportion of the adult population presenting at least one risk factor was estimated using prevalence data of the considered risk factors taken from local health surveys, official country statistics and published literature. These estimates were corrected to limit double counting of people with multiple risk factors.^18-20^ Population size estimates were based on 2014 country official statistics^21-23^ (see Table 1).

**Table 1.** Age-specific model inputs, by age group and country.

	Brazil				Colombia				Panama			
Parameter	Young children (6–59 months)	Adults 18–49 y with RF	Adults 50–59 y with RF	Elderly (60 y and older)	Young children (6–23 months)	Adults 18–49 y with RF	Adults 50–59 y with RF	Elderly (60 y and older)	Young children (6–59 months)	Adults 18–49 y with RF	Adults 50–59 y with RF	Elderly (60 y and older)
Population for whom vaccination is recommended (% of the age-specific country population)	13,480,426 (100%)	27,721,888 (27.6%)	8,898,508 (42.5%)	22,988,618 (100%)	1,300,446 (100%)	4,611,562 (20.7%)	1,436,143 (30.2%)	5,146,251 (100%)	331,615 (100%)	420,176 (22.8%)	117,298 (32.4%)	416,433 (100%)
Vaccine coverage rate	91.2%	17.3%	23.5%	87.0%	67.4%	28%^a^	28%^a^	28.0%	49.9%	97.4%	97.4%	76.7%
**Vaccine effectiveness**												
Against influenza A (TIV, QIV)	59%	61%	61%	59%	59%	61%	61%	59%	59%	61%	61%	59%
Against matched B lineage (TIV, QIV)^b^	66%	77%	73%	70%	66%	77%	73%	70%	66%	77%	73%	70%
Lineage cross-protection, % of matched B effectiveness (TIV)	67%	68%	67%	68%	67%	68%	67%	68%	67%	68%	67%	68%
Average influenza attack rate over the study period	18.8%	3.6%	3.6%	4.5%	18.8%	3.6%	3.6%	4.5%	18.8%	3.6%	3.6%	4.5%
**Resource use and outcomes**												
Influenza-related GP consultation, % per influenza infection^c^	47.9%	62.6%	62.6%	63.2%	40.5%–63.0%	54.8%–82.4%	44.1%–82.4%	15.9%–87.0%	51.1%–63.0%	47.3%–82.4%	48.6%–82.4%	51.2%–87.3%
Working days lost, per GP consultation	0.85	1.35	2.74	1.66	0.88	1.39	2.94	1.33	0.89	1.42	2.87	1.54
Influenza-related hospitalizations, per 1,000 influenza infections^c^	14.1	4.2	19.3	34.7	25.2–214.8	2.0–64.0	3.5–294.1	13.1–529.5	3.7–214.8	0.8–64.0	1.8–294.1	9.5–536.3
Influenza related deaths, per 1,000 influenza infections^c^	0.04	0.09	1.34	8.32	0.11–1.05	0.14–2.35	2.61–35.04	4.12–218.63	0.15–1.24	0.08–2.79	0.27–41.48	2.66–265.08
**Unit costs**^d^												
Influenza-related GP consultations	BRL 10	BRL 10	BRL 10	BRL 10	COP 54,978	COP 45,923	COP 59,986	COP 84,549	PAB 12	PAB 12	PAB 12	PAB 12
Influenza-related hospitalizations	BRL 1,069	BRL 909	BRL 1,640	BRL 1,432	COP 1,095,349	COP 2,666,209	COP 3,517,367	COP 4,004,381	PAB 755	PAB 973	PAB 1,191	PAB 1,332
Productivity loss, per working day lost	BRL 72	BRL 72	BRL 97	BRL 110	COP 20,533	COP 20,533	COP 20,533	COP 20,533	PAB 26	PAB 26	PAB 26	PAB 26

GP: General Practitioner, QIV: Quadrivalent influenza vaccine; TIV: Trivalent influenza vaccine; RF: Risk factor, namely: pregnant women and people suffering from pulmonary diseases (asthma, COPD), cardiovascular diseases, diabetes mellitus, chronic renal disease, hepatic diseases, or HIV/AIDS.

a assumed similar to coverage rate in the elderly.

b QIV effectiveness is assumed to be the same for both matched and mismatched B lineages.

c Inputs for Colombia and Panama are presented as a range corresponding to 2 scenarios using different sources.

d In local currency 2014; 2014 average exchange rates from World Bank: US$ 1 = BRL 2.35, US$ 1 = COL 2,002, US$ 1 = PAB 1.

### Influenza circulation

Estimation of the seasonal influenza attack rate is generally not straightforward,^24^ and influenza surveillance data in Latin American countries were scarce. Thus in order to generate a strain- and lineage-specific attack rate by season for each of the 3 countries, we adopted the same, stepped method for each of the 3 countries.

First, age-specific average annual attack rates in unvaccinated populations were retrieved from the influenza incidence rates in pooled control arms of clinical trials presented in 3 Cochrane reviews for healthy children, healthy adults and the elderly (Table 1).^25-27^ These rates were then distributed across seasons for each country using the following formula: $AR_{season,group}=\;AR_{group}\times \beta_{season}/(Average\left(\beta_{season}\right),$ where $AR_{season,\;\;group}$ denotes the age- and season-specific influenza attack rate, $AR_{group}$ denotes the average attack rate for the given age group and $\beta_{season}$ denotes the season-specific intensity coefficient. This coefficient was defined differently for the 3 countries. On the one hand, intensity coefficients for Brazil were defined as the number of influenza specimens which were tested positive as reported by influenza surveillances programs in Sao Paulo state,^28,29^ which was considered to have the more robust data in Brazil in terms of influenza surveillance and on the other hand, intensity coefficients for Colombia and Panama were defined as the proportion of positive influenza specimens among all specimens reported for each country in the FluNet database.^30^

Second, the season-specific attack rates were split according to the influenza strain distribution (strains A vs B) in each country and in each season. The proportions of strain A among all influenza strains were derived from the number of influenza specimens reported in the same sources used for the season-specific intensity coefficients.

Third, the attack rate attributable to the B strain was further split according to the influenza B lineage distribution (Yamagata or Victoria). Data on distribution of B lineages among all B strains were scarce, and we identified data from characterized influenza specimens analyzed in the Sao Paulo state during the period 2002–2014^10,31^ to be the most reliable source for distribution of B lineage in the 3 countries. As a consequence, the distribution of B lineages (Victoria versus Yamagata) among all B strains was assumed to be similar for the 3 countries except for 2014 when local FluNet data were available for Colombia and Panama (Table 2). The year 2014 was thus the only point of comparison between the 3 countries, during which the Yamagata lineage represented 93%, 40% and 100% of the characterized B strains in Brazil, Colombia and Panama, respectively.

**Table 2.** Influenza circulation and B lineage included in TIV by country and by season.

		Brazil					Colombia					Panama				
		Distribution of influenza circulation by strain and B lineage (% (N)))					Distribution of influenza circulation by strain and B lineage (% (N))					Distribution of influenza circulation by strain and B lineage (% (N))				
Season	B lineage included in TIV^a^	A^e^	B/Victoria^f^	B/Yamagata^f^	B mismatch^b^	Season intensity coefficient^c^	A^e^	B/Victoria^f^	B/Yamagata^f^	B mismatch^b^	Season intensity coefficient^d^	A^e^	B/Victoria^f^	B/Yamagata^f^	B mismatch^b^	Season intensity coefficient^d^
2006	Victoria											95.9%^g^ (47)	2.7% (4)	1.4% (2)	Medium	13.9%
2007	Victoria						87.0% (20)	8.7% (2)	4.3% (1)	Medium	1.4%	73.2% (52)	17.8% (2)	8.9% (1)	Medium	11.6%
2008	Yamagata						75.0% (6)	3.9% (5)	21.1% (27)	Low	1.8%	78.7% (85)	3.3% (5)	18.0% (27)	Low	8.9%
2010	Victoria	83.9% (952)	0.0% (0)	16.1% (3)	Complete	1,135	93.0% (731)	0.0% (0)	7.0% (3)	Complete	7.2%	93.2% (193)	0.0% (0)	6.8% (3)	Complete	13.3%
2011	Victoria	84.4% (847)	15.6% (1)	0.0% (0)	Null	1,003	98.9% (610)	1.1% (1)	0.0% (0)	Null	6.0%	100.0% (44)	0.0% (1)	0.0% (0)	Null	3.7%
2012	Victoria	90.6% (1,430)	3.1% (1)	6.3% (2)	High	1,579	92.5% (617)	2.5% (1)	5.0% (2)	High	5.8%	30.2% (62)	23.3% (1)	46.5% (2)	High	11.5%
2013	Yamagata	74.0% (5,879)	23.1% (16)	2.9% (2)	High	7,943	96.0% (864)	3.6% (16)	0.4% (2)	High	9.6%	100.0% (188)	0.0% (16)	0.0% (2)	Null	8.8%
2014^h^	Yamagata	84.5% (547)	1.2% (4)	14.3% (49)	Low	1,790	76.0% (494)	14.5% (94)	9.5% (62)	Medium	9.6%	61.0% (86)	0.0% (0)	39.0% (55)	Null	7.7%

a Common to Brazil, Colombia and Panama

b Null: 0% Low: <33%, Medium: [33%; 66%], High: >66%, Complete: 100%.

c Number of influenza-positive specimens in Sao Paulo surveillance network.

d Colombia and Panama: Proportion of positive specimens among all tested specimens reported in FluNet weekly reports.

e (N) corresponds to the number of specimens positive to influenza A used to derive the proportion of A among all strains.

f (N) corresponds to the number of characterized B specimens used to derive the proportion of B lineages among B strains.

g Colombian FluNet data for proportion of A were used for Panama in 2006 as there was no local data available. As the TIV was launched in 2007 in Colombia, the season 2006 was not considered in the analysis.

h Local FluNet data were used for Colombia and Panama for B lineage distribution in 2014.

### Vaccine coverage and effectiveness

Age-specific vaccine effectiveness of TIV against matched B lineage and mismatched B lineage was approximated by the vaccine efficacy of inactivated TIV, as reported in Clements et al.^32^ The estimates considered by Clements et al. were extrapolated from a meta-analysis^33^ in which B lineage cross-protection was found to be approximately 68% of the effectiveness against the matched B lineage in adults (vaccine efficacy was found to be 77% against matched B lineage and 52% against mismatched B lineage), which is in line with the conclusions of a review on the efficacy of influenza vaccines by Diaz Granados, Denis and Plotkins.^34^ A more recent study^35^ estimated that the vaccine effectiveness of TIV when the Yamagata lineage was included, was overall 66% (95%CI: 58–73%) against Yamagata lineage, vs. 51% against Victoria (95%CI: 36–63%). However, these data were estimated on a single influenza season and do not dramatically differ from the estimates used in Clements et al.^32^ Moreover, the range of estimates observed in the literature is covered in the sensitivity analyses.

QIV efficacies for both B lineages were assumed to be the same as the inactivated TIV efficacy against the matched B lineage (Table 1). B lineages contained each year in the TIV and corresponding to the southern hemisphere formulation for the 3 countries were retrieved from the WHO annual recommendations.^36^

Coverage rates for the population groups of each country were estimated from local sources. Brazilian coverage rates were retrieved from the DATASUS database^37^ as an average of years 2013 and 2014. Colombian rates were retrieved from 2013 data provided by the Colombian Ministry of Health and Social Protection. Due to a lack of data, Colombian adults with risk factors were assumed to have the same coverage rate as the elderly. Finally, Panamanian coverage rates were computed as the average coverage rates for the years 2010 to 2013, retrieved from data published by the Panamanian Ministry of health.

### Influenza outcomes

In the absence of relevant data sources regarding the healthcare resource use and outcomes associated with an influenza infection for Brazil, we decided to consider data from a US cost of illness study^38^ for the probabilities of outpatient visits, hospitalizations and deaths following influenza infection (see Table 1). Hospitalization and death rates were calculated based on excess rates of hospitalizations and deaths that were reported to be due to respiratory and circulatory conditions, using peri-seasonal risk-difference models.^38^ Although the methods of estimation of these US rates were robust, considering the US hospitalization rates might lead to overestimate the Brazilian rates in the case where health care access would be lower for influenza related outcomes in Brazil. In absence of local data, this choice was found reasonable and rates were varied in sensitivity analyses.

In Colombia and Panama, we considered 2 sets of inputs for the rates of GP consultations, hospitalizations and deaths attributable to influenza which corresponded to low and high estimates of the influenza burden.

The inputs for the first scenarios were estimated using local databases. In Colombia, rates of outpatient visits, hospitalizations and deaths related to influenza were collected using 2013 data from an insurance claims database of a Colombian Health Promotion Agency (EPS)^39^ representing more than 3 million affiliates. In Panama, GP visit rates were computed from 2010 to 2013 by considering the influenza-coded consultations in an ambulatory database.^40^ Hospitalization rates were derived for 2011–2013 from an inpatient database^41^ where all hospitalizations coded as influenza and pneumonia were considered, and assuming that 8.6%^42^ of these hospitalizations were attributable to influenza. A similar approach was taken for mortality rates where we considered that 8.5%^43^ of deaths retrieved from annual mortality reports^44^ from 2007 to 2013 and coded as due to influenza or pneumonia were really attributable to influenza. Although these correction factors come from US studies, we preferred using robust US data that were obtained using peri-seasonal risk difference models rather than other extrapolations such as correcting by the proportion of influenza positive specimen from influenza circulation data which would certainly overestimate the influenza burden.

In the second scenario, we took values for GP consultation, hospitalization and mortality rates from 2 Colombian studies,^45,46^ which were extrapolated to the age groups of analysis using US data from Molinari et al.^38^

For the 3 countries and for all scenarios, the GP consultation rates for adults with risk factors were assumed to be twice as high as the rates for the standard risk population, just as in Molinari et al.^38^ It was not possible to differentiate hospitalizations and mortality rates by risk status in the general adult population, which underestimated the influenza burden for adults with risk factors. Lastly, we considered that patients who consulted a GP due to an influenza infection would be associated with productivity loss due to work absenteeism. For children, the number of working days lost by the caregivers was taken into account. The age-specific number of working days lost per GP consultation was retrieved from Molinari et al. and adjusted with the country-specific employment rates.^47-49^

### Economic inputs

We considered the public costs of GP consultations and hospitalizations estimated from a cost database for Brazil^37,50^ while Colombian unit costs were retrieved from the same insurance claims database that was used to estimate the influenza-related outcome rates.^39^ In the absence of recent data sources for estimating Panamanian costs, we considered the WHO 2008 unit costs^51^ of GP consultations and hospitalizations which are not specific to influenza. We adjusted the latter with the influenza-related cost of hospitalization in the elderly derived from Chit et al.,^52^ and combined it with the number of hospitalization days by age group from Thompson et al.^42^ All unit costs were calculated in 2014 local currencies and were inflated using the health component of the Consumer Price Index when needed.

The productivity loss associated with a working day lost in each of the 3 countries was valued as the average daily wage in 2014 retrieved from official statistics, assuming an average of 22 workdays in a month. Furthermore, we conservatively assumed that deaths were not associated with any cost.

## Sensitivity analyses

In order to explore the impact of uncertainty in the input parameters on the additional influenza burden that could be avoided by QIV, we conducted deterministic sensitivity analyses on the additional number of influenza cases avoided and associated societal costs avoided in Brazil over the period 2010–2014. The list of parameters included in the sensitivity analyses and their tested values are presented in Supplementary file 1.

Given that the key parameters were the same for Colombia and Panama, we might expect similar variations in the results. However, as there was already high uncertainty in the influenza outcomes, influenced by 2 separate sets of inputs, we did not present additional sensitivity analyses for these 2 countries.

## Results

### Base case

In Brazil over the period 2010–2014, the replacement of TIV by QIV for the whole population included in the analysis (n = 73,089,440) was estimated to prevent more than 615,000 additional influenza cases, associated with a reduction of 326,500 GP consultations, 11,730 hospitalizations and 1,385 deaths (see Table 3). The public health impact would have been associated with TPP influenza-related cost offsets of 18 million Brazilian reals while productivity loss would have accounted for 2 thirds of the BRL 53 million of societal costs avoided (equivalent to US$ 23 million or US$ 6,200 per 100,000 person-years). Young children would have benefited the most from the QIV introduction in terms of influenza cases, GP consultations and hospitalizations avoided, with 599 cases, 287 consultations and 8.4 hospitalizations avoided per 100,000 person-years (see Table 4). The elderly would have also benefited substantially from the introduction of QIV with 4.9 hospitalizations and 1.17 deaths avoided per 100,000 person-years. Adults with risk factors would have had fewer benefits over the period with 25 and 33 influenza cases avoided per 100,000 person years for 18–49 y old and 50–59 y old, respectively. In Colombia, between the introduction of influenza vaccination in 2007, and the year 2014 (2009 excluded), it was estimated that using QIV instead of TIV would have avoided about 29,700 additional influenza cases in the total population of interest, leading to a potential reduction of between 11.2 and 21.6 thousand GP consultations, between 510 and 7,775 hospitalizations, and between 34 and 1,524 deaths. Economically, QIV was estimated to lead to societal cost offsets of between COP 1.7 billion and COP 22.2 billion over the period 2007–2014 (between US$ 868,000 and US$ 11,077,000, or between US$ 1000 to US$ 12,700 per 100,000 person-years), mostly driven by direct medical costs which represented 84% to 98% of total costs. The costs avoided were highest for young children (US$ 5,100 to US$ 25,100 in additional costs avoided per 100,000 person-years).

**Table 3.** Total influenza-related events and associated costs (in 2014 local currencies) avoided by using QIV instead of TIV in the population of analysis over the study period.

	Brazil (2010–2014)	Colombia^*^ (2007–2014, 2009 excluded)	Panama^*^ (2006–2014, 2009 excluded)
Outcomes			
Influenza cases avoided	615,040	29,665	11,582
GP consultations avoided	326,494	11,204–21,607	5,650–8,424
Lost working days avoided	389,380	13,235–26,487	7,318–11,416
Hospitalizations avoided	11,732	511–7,775	47–2,861
Deaths avoided	1,385	34–1,524	8–706
Influenza-associated costs			
GP consultation costs avoided	BRL 3,264,939	COP 624–1,325 million	PAB 66,827–99,645
Hospitalization costs avoided	BRL 14,715,777	COP 841–20,307 million	PAB 50,177–3,067,384
Direct costs avoided (Third-Party Payer perspective)	BRL 17,980,716	COP 1,465–21,631 million	PAB 117,004–3,167,029
Productivity loss	BRL 35,265,257	COP 272–544 million	PAB 191,123–298,149
Total costs avoided (Societal perspective)	BRL 53,245,973	COP 1,737–22,175 million	PAB 308,127–3,465,178

GP: General practitioner

* Colombia and Panama: results are presented as a range using the 2 sets of values presented in Table 1.

**Table 4.** Average influenza-related events and associated costs (in 2014 US$) avoided by using QIV instead of TIV over the study period, per 100,000 person-years.

Brazil (2010–2014)					
	Young children (6–59 months)	Adults 18–49 y with RF	Adults 50–59 y with RF	Elderly (60 y and older)	Total
Outcomes					
Influenza cases avoided	599	25	33	141	168
GP consultations avoided	287	16	20	89	89
Lost working days avoided	244	21	56	148	107
Hospitalizations avoided	8.4	0.1	0.6	4.9	3.2
Deaths avoided	0.02	0.00	0.04	1.17	0.38
Influenza-associated costs					
Direct costs avoided (Third-Party Payer perspective)	$5,065	$108	$527	$3,353	$2,094
Productivity loss	$7,529	$654	$2,319	$6,955	$4,106
Total costs avoided (Societal perspective)	$12,594	$761	$2,845	$10,308	$6,200
Colombia (2007–2014, 2009 excluded)					
	Young children (6–23 months)	Adults 18–49 y with RF	Adults 50–59 y with RF	Elderly (60 y and older)	Total
Outcomes					
Influenza cases avoided	178	16	16	18	34
GP consultations avoided	72–112	9–13	7–13	3–16	13–25
Lost working days avoided	63–99	12–19	20–38	4–21	15–30
Hospitalizations avoided	4.5–38.3	0.0–1.0	0.1–4.6	0.2–9.7	0.6–8.9
Deaths avoided	0.02–0.19	0.00–0.04	0.04–0.55	0.08–4.00	0.04–1.74
Influenza-associated costs					
Direct costs avoided (Third-Party Payer perspective)	$4,445–$24,067	$248–$1,699	$305–$8,477	$601–$20,027	$837–$12,355
Productivity loss	$651–$1,014	$128–$192	$208–$389	$40–$217	$155–$311
Total costs avoided (Societal perspective)	$5,096–$25,081	$376–$1,891	$513–$8,866	$641–$20,245	$992–$12,666
Panama (2006–2014, 2009 excluded)					
	Young children (6–59 months)	Adults 18–49 y with RF	Adults 50–59 y with RF	Elderly (60 y and older)	Total
Outcomes					
Influenza cases avoided	201	86	83	77	113
GP consultations avoided	100–124	40–70	39–67	38–65	55–82
Lost working days avoided	90–111	57–99	113–192	59–101	71–111
Hospitalizations avoided	0.7–42.2	0.1–5.4	0.1–23.8	0.7–40.1	0.5–27.8
Deaths avoided	0.03–0.24	0.01–0.23	0.02–3.36	0.20–19.82	0.08–6.87
Influenza-associated costs					
Direct costs avoided (Third-Party Payer perspective)	$1,744–$33,353	$538–$6,075	$638–$29,156	$1,401–$54,162	$1,138–$30,795
Productivity loss	$2,349–$2,896	$1,479–$2,580	$2,956–$5,009	$1,542–$2,629	$1,858–$2,899
Total costs avoided (Societal perspective)	$4,093–$36,250	$2,017–$8,655	$3,593–$34,165	$2,942–$56,791	$2,996–$33,694

RF: Risk factor

* Colombia and Panama: results are presented as a range using the 2 sets of values presented in Table 1.

In Panama over the period 2006–2014 (2009 excluded), it was estimated on average that QIV would have prevented 11,600 influenza cases, between 47 and 2,860 hospitalizations, and between 8 and 706 deaths. This would have translated into total costs avoided of between PAB 308,000 and PAB 3,465,000 from the societal perspective. In the low case scenario, estimated costs offsets were mostly due to productivity loss (62%) while 92% of societal costs were due to hospitalization costs in the high case scenario.

**Figure 2.**
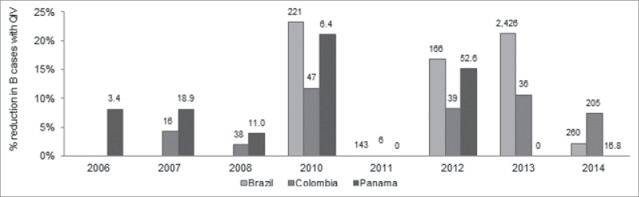
Reduction in B influenza cases associated to QIV compared with TIV by year and country of analysis., The number of B cases occurring in the TIV scenario (in thousands of cases) is displayed at the top of each bar. There was no impact of QIV in 2011 as the source used for B lineage distribution did not allow for a precise estimate (only one specimen was tested which corresponded to the lineage included in TIV). Interpretation: In 2012 in Panama, there were 52,600 cases of influenza B cases despite the use of TIV while there were 15% less influenza B cases with QIV., QIV: Quadrivalent influenza vaccine; TIV: Trivalent influenza vaccine.

For instance in 2013, QIV would have reduced the number of B cases by 22% (out of a total number of B cases of more than 2.4 million) in Brazil and by 13% (out of 36 thousand B cases) in Colombia with an absence of B circulation in Panama during this year (see Fig. 2). QIV would have had the most impact in 2013 in Brazil, with 528,000 additional influenza cases avoided, 2014 in Colombia with more than 15,500 additional cases avoided and in 2012 in Panama with 8,000 cases avoided. Overall, seasons 2010 and 2012 would have associated with substantial QIV benefits for the 3 countries with a reduction in the number of B cases ranging from 8.2% (Colombia, 2012) to 23.3% (Brazil, 2010), compared with TIV.

### Sensitivity analyses

**Figure 3.**
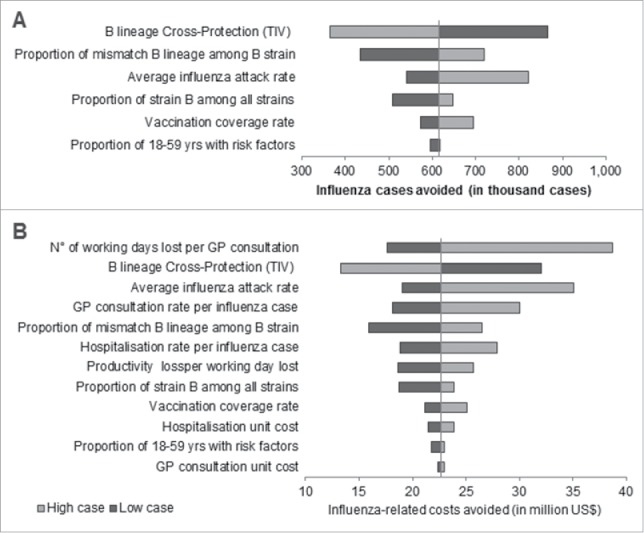
Deterministic sensitivity analyses results performed on Brazil for the total recommended population over the period 2010–2014 on A) Influenza cases avoided and B) total influenza-related societal costs avoided (in US$), GP: General Practitioner; QIV: Quadrivalent influenza vaccine; TIV: Trivalent influenza vaccine.

Results of the sensitivity analyses for Brazil over 2010–2014 are presented in Fig. 3. The level of cross protection of TIV against mismatched B lineage was identified as the parameter with the greatest impact on the number of influenza cases avoided with between 365,000 and 865,000 influenza cases avoided when varying the degree of cross protection by ± 20%. Uncertainty in influenza circulation parameters had also substantial impact on the number of additional influenza cases avoided, preventing from 433,000 cases when considering low proportions of mismatch B lineage to 821,000 cases when considering the higher bound of the average annual influenza attack rate. The level of cross-protection also translated to high uncertainty in terms of influenza-related societal costs avoided with estimated costs avoided ranging from US$ 13 to 32 million. The associated societal costs were also sensitive to the number of working days lost per influenza consultation with up to US$ 39 million of societal costs avoided when considering the high case values. For most parameters, uncertainty around parameters led to additional societal costs avoided ranging between US$ 16 to US$ 30 million over the period 2010–2014.

## Discussion

In recent years the interest in quadrivalent influenza vaccines has grown as TIV only matches the predominantly circulating B lineage while predicting which of the 2 B lineages will be circulating remains a challenge. We modeled the impact of QIV compared with TIV vaccination in 3 Latin American countries over a number of past influenza seasons based on the same approach as Reed et al.^11^ and Lee et al.^12^ In our study, we estimated that the use of QIV instead of TIV would have reduced the clinical burden of influenza, avoiding between 13 to 89 GP consultations, 0.5 to 27.8 hospitalizations and 0.04 to 6.87 deaths per 100,000 person-years over the study population, depending on the country. The public health benefits brought by QIV would have been equivalent to between US$ 1,000 and US$ 34,000 in societal costs per 100,000 person-years. Considering the most conservative parameter values from the sensitivity analyses, it was estimated that QIV would have prevented 365,000 influenza cases over 5 y with associated cost offsets equivalent to US$ 13.3 million in Brazil.

In Brazil, it was estimated that the young children and the elderly would have been the subgroups that would have benefited the most from QIV introduction, this could be explained by a substantially lower vaccine coverage rate (18–26%) for these groups compared with young children (91%) and the elderly (87%), a lower average influenza attack rate in these age groups, and by undifferentiated hospitalization and mortality rates relative to their risk status.

In Colombia, there was substantial variation between the estimates of the numbers of hospitalizations, and deaths avoided, due to high uncertainty in the probability of hospitalization and death following influenza infection, with the true impact of QIV being likely to lie within this range. Furthermore, due to a lower vaccine coverage rate in people older than 60 y compared with the 2 other countries, the benefits of QIV for the elderly compared with young children were significantly lower for Colombia than for Brazil and Panama.

Although the degree of mismatch across influenza seasons was considered identical for the 3 countries for most of the seasons, the impact of QIV by year varied between countries due to differences in coverage rates and the intensity of each season. In 2011, there was no impact of QIV estimated as there was only one characterized influenza B specimen which had its lineage tested for this season, leading to a 100% match. Before the season 2010, impact of QIV would have been limited in Colombia and Panama due to low B circulation and mismatch.

Compared to the studies by Reed et al.^11^ and Lee et al.^12^ 2 enhancements were made to the model. First, our analysis accounted for the efficacy of TIV against mismatched B lineage, which was assumed to be null by Reed et al. Secondly, the population was stratified into 3 groups among those at the highest risk of influenza complications so as to better reflect the differences in vaccination coverage, vaccine effectiveness and risks of complications between groups. The stratification was important especially because the vaccine effectiveness is lower among the elderly, who also have a higher risk of complications compared with other population groups.

In the US, Reed et al. estimated that 0.72 hospitalizations per 100,000 person-years would have been avoided during the seasons 1999/2000 to 2008/2009. This estimate is included in the estimated range for Colombia and Panama, which is lower than the rate estimated for Brazil (3.3 hospitalizations per 100,000 person years). This difference can be explained by several factors. Among which are: that the population included in our study was more susceptible to influenza complications than the general US population since we did not take into account people with low risk of influenza complications; in terms of the economic consequences of the introduction of QIV, Lee et al. included lifetime productivity loss associated with influenza mortality; combined with the fact that our analysis was performed in countries with lower healthcare costs and lower wages than in the US. This explains the substantially higher costs avoided estimated by Lee et al., compared with our estimates: about US$ 110,000 per 100,000 persons from the societal perspective in the US, vs. US$ 380 to US$ 56,800 in our study, depending on subgroup and country.

Our analysis suffers from several limitations. There were data gaps regarding the circulation of the 2 B lineages and the burden of influenza in Latin American countries. Estimates of the Sao Paulo state were used to inform the B lineage circulation in the 3 countries. Although these estimates come from a region with a well performing surveillance system and that similar distributions were found when using other sources in Brazil^7^ the number of tested specimens remained low, translating into high statistical uncertainty. Furthermore, by using Brazilian data, we implicitly assumed a similar influenza circulation between the 3 countries, which was considered reasonable since Brazil and Colombia belong to the “Temperate South America” influenza transmission zone while Panama is located just outside of the frontier of this transmission zone. The potential impact of the uncertainty around influenza B circulation was tested in the deterministic sensitivity analyses through a broad range of inputs. Although there was uncertainty around the B lineage distribution in the 3 countries, 2 specificities of our analysis reduces the potential effects on the estimated impact of QIV: the proportion of A strain among all strains were taken from local sources in the 3 countries, limiting the uncertainty to the number of B cases, and performing the analysis on several years (5 for Brazil, 8 for Panama, 7 for Colombia) limited the risk of substantially underestimating or overestimating the overall B mismatch in these countries by reflecting the variations in influenza circulation from one season to another.

In the absence of reliable data for the influenza burden in Brazil, we used robust foreign data from a US study. This choice had inherent limitations since the characteristics of the Brazilian healthcare system and population are different from those of the US. For instance, the mean number of doctor consultations was estimated to be 4.1 per capita in the US as against 2.7 per capita in Brazil.^53^ This variation in the propensity for consulting a doctor was also tested in sensitivity analyses. Another approach was taken for Colombia and Panama by considering 2 scenarios using local data and other sources. This led to a high uncertainty in the influenza burden avoided in these countries, with the true impact of QIV likely to lie within this range. Further developments in influenza surveillance systems as well as in healthcare databases would be needed to assess the true impact of influenza more accurately, and thus the impact of QIV in Latin America.

Another limitation of the influenza models is that the influenza distributions were not considered to be age-specific. Thus, although we considered age-specific attack rates and despite the fact that influenza B places a disease burden on all age groups, its incidence relative to influenza A appears to be highest among older children and young adults,^9^ a population with a lower risk of complications. There was no distinction made between the severity of A and B strains either, because so far the literature has not shown significant differences in the clinical burden between influenza A and influenza B.^54^

From an economic stance, estimated influenza-related costs avoided estimated did not reflect the entire economic burden avoided. Medical costs of death, transportation costs as well as potential differences in costs based on the setting (private/public) were not taken into account, as well as productivity loss associated with premature deaths and hospitalizations. These choices were made to provide conservative estimates of the impact of QIV in a context of high uncertainty around the healthcare costs of influenza.

Although uncertainty around the distribution of B lineage and the true influenza burden was high in Latin America, we think the results of this study is an essential step for the estimation of the benefits of QIV. First, to date, there are no published studies assessing quantitatively the impact of the introduction of QIV in Latin America. Recently, 2 studies, Barros et al.^7^ and Arlant and Bricks,^55^ summarized evidence corroborating our data collection on the circulation of B lineage. Our study is thus the first one to provide quantitative estimates of the benefits of vaccination with QIV instead of TIV. Second, we considered several points to reduce the impact of uncertainty around results. We estimated the impact of QIV using data circulation from several seasons which allowed taking into account differences in influenza circulation from year to year. We also used robust local data from the FluNet surveillance network which contained hundreds of analyzed specimens for most of the years included in the analysis to estimate the distribution between A and B strains, which mechanically reduced the uncertainty around the influenza B burden. Last, if influenza burden attributable to each B lineage for a particular year might differ from the reality due to data uncertainty, the aggregated burden over several years is likely to be close to the true burden. Indeed, published studies exploring influenza circulation in many parts of the world showed similar trends as what was seen in our study. For instance, Caini et al.,^56^ using data from the period 2000–2013 in 26 countries, showed that 20–30% of influenza cases were from the B strains, a number similar to our study (12% in Colombia, 17% in Brazil, 21% in Colombia). Furthermore, the same study showed B lineages co-circulated in a large proportion of seasons, with Victoria predominating more often than Yamagata (64% against 36%), similarly to our study.

Finally, some additional analyses and improvements of the model should be considered in future research. Firstly, vaccination costs were not taken into account in our study because we focused mainly on the medical costs and loss of productivity related to influenza. A cost-effectiveness analysis could be conducted by taking into consideration the vaccination costs associated with QIV and TIV. Secondly, because of the scarcity of local data, we used a static model to estimate the impact of QIV. Static models are unable to account for changes in the force of infection arising from the reduction in the prevalence of infectious individuals that can be brought by vaccination or acquired immunity.^57^ These models are only able to capture the impact of direct protection at the very start of an influenza season, resulting potentially, in the underestimation of the benefits of vaccination, compared with dynamic models. In addition, static models do not take into account different contact rates between individuals according to their age or social characteristics, which have an impact on the transmission of influenza strains across population groups. However, as dynamic modeling is a complex approach which requires extensive data, subject to data availability, a further step could be to refine the estimation of QIV benefits using a dynamic model.

## Supplementary Material

KHVI_A_1256928_Supplementary_material.zip

## Abbreviations

BRL: Brazilian Real
COP: Colombian Peso
GP: General Practitioner
PAB: Panamanian balboa
QIV: Quadrivalent influenza vaccine
TIV: Trivalent influenza vaccine
TPP: Third-Party payer
WHO: World Health Organization

## References

[1] Centers for Disease Control and Prevention (CDC) People at high risk of developing flu–related complications. 2015 [Cited 01/03/2016]. Available from: http://www.cdc.gov/flu/about/disease/high_risk.htm

[2] Harper SA, Fukuda K, Uyeki TM, Cox NJ, Bridges CB Prevention and control of influenza: recommendations of the Advisory Committee on Immunization Practices (ACIP). MMWR Recomm Rep 2004; 53:1-40; PMID:15163927 15163927

[3] Savy V, Ciapponi A, Bardach A, Glujovsky D, Aruj P, Mazzoni A, Gibbons L, Ortega-Barria E, Colindres RE Burden of influenza in Latin America and the Caribbean: a systematic review and meta-analysis. Influenza Other Respir Viruses 2013; 7:1017-32; PMID:23210504; http://dx.doi.org/10.1111/irv.12036 23210504 PMC4634294

[4] Centers for Disease Control and Prevention (CDC) Vaccine Effectiveness - How well does the flu vaccine work ? 2015 [Cited 01/03/2016]. Available from: http://www.cdc.gov/flu/about/qa/vaccineeffect.htm

[5] World Health Organization (WHO) Influenza Fact sheet n 211. 2014 2014 [Cited 01/03/2016]. Available from: http://www.who.int/mediacentre/factsheets/fs211/en/

[6] Ropero-Alvarez AM, El Omeiri N, Kurtis HJ, Danovaro-Holliday MC, Ruiz-Matus C Influenza vaccination in the Americas: Progress and challenges after the 2009 A(H1N1) influenza pandemic. Hum Vaccin Immunother 2016; 12:2206-14; PMID:27196006; http://dx.doi.org/10.1080/21645515.2016.1157240 27196006 PMC4994725

[7] Barros EN, Cintra O, Rossetto E, Freitas L, Colindres R Patterns of influenza B circulation in Brazil and its relevance to seasonal vaccine composition. Braz J Infect Dis 2016; 20:81-90; PMID:26626166; http://dx.doi.org/10.1016/j.bjid.2015.09.009 26626166 PMC7110561

[8] Belshe RB The need for quadrivalent vaccine against seasonal influenza. Vaccine 2010; 28 Suppl 4:D45-D53; PMID:20713260; http://dx.doi.org/10.1016/j.vaccine.2010.08.028 20713260

[9] Ambrose CS, Levin MJ The rationale for quadrivalent influenza vaccines. Hum Vaccin Immunother 2012; 8:81-8; PMID:22252006; http://dx.doi.org/10.4161/hv.8.1.17623 22252006 PMC3350141

[10] Carvalhanas TRMP, Paiva TM, Benega MA, Silva DBB, Paulino RS, Santos KCO, Ferreira PM, Yu ALF, Pinto FKA, Bricks LF Influenza circulation in Sao Paulo state (SP), Brazil: review of 2 seasons (2013-2014). 33rd Annual Meeting of the European Society for Paediatric Infectious Diseases 2015; Leipzig, Germany European Society for Paediatric Infectious Diseases.

[11] Reed C, Meltzer MI, Finelli L, Fiore A Public health impact of including two lineages of influenza B in a quadrivalent seasonal influenza vaccine. Vaccine 2012; 30:1993-8; PMID:22226861; http://dx.doi.org/10.1016/j.vaccine.2011.12.098 22226861

[12] Lee BY, Bartsch SM, Willig AM The economic value of a quadrivalent versus trivalent influenza vaccine. Vaccine 2012; 30:7443-6; PMID:23084849; http://dx.doi.org/10.1016/j.vaccine.2012.10.025 23084849 PMC3696129

[13] The World Bank. Official exchange rate (LCU per US$, period average). 2014 [Cited 10/05/2016]. Available from: http://data.worldbank.org/indicator/PA.NUS.FCRF

[14] Brasil. Ministério da Saúde. Informe Técnico. Campanha Nacional de Vacinação contra Influenza. 2015. Available from: http://portalsaude.saude.gov.br/images/pdf/2015/abril/09/Informe-Cp-Influenza—25-03-2015-FINAL.pdf [Accessed 14/09/2016].

[15] Panama. Ministerio de Salud. Comision Nacional Asesora de Practicas de inmunizaciones. Esquema Nacional de Vacunación. 2013. Available from: http://www.minsa.gob.pa/sites/default/files/programas/esquema_de_vacunacion_revisado_marzo_2013.pdf [Accessed 14/09/2016].

[16] Colombian Ministry of Health and Social Protection Vacuna against influenza. 2013 [Cited 01/03/2016]. Available from: https://www.minsalud.gov.co/salud/Documents/Influenza.pdf

[17] World Health Organization WHO position paper on influenza vaccines – August 2005. Weekly Epidemiological Record (WER) 2005; 80:277-88.16171030

[18] Balzi D, Barchielli A, Buiatti E, Franceschini C, Lavecchia R, Monami M, Santoro GM, Carrabba N, Margheri M, Olivotto I, et al. Effect of comorbidity on coronary reperfusion strategy and long-term mortality after acute myocardial infarction. Am Heart J 2006; 151:1094-100; PMID:16644342; http://dx.doi.org/10.1016/j.ahj.2005.06.037 16644342

[19] Longmore RB, Spertus JA, Alexander KP, Gosch K, Reid KJ, Masoudi FA, Krumholz HM, Rich MW Angina frequency after myocardial infarction and quality of life in older versus younger adults: the prospective registry evaluating myocardial infarction: event and recovery study. Am Heart J 2011; 161:631-8; PMID:21392621; http://dx.doi.org/10.1016/j.ahj.2010.12.005 21392621

[20] van der Molen T Co-morbidities of COPD in primary care: frequency, relation to COPD, and treatment consequences. Prim Care Respir J 2010; 19:326-34; PMID:20842323; http://dx.doi.org/10.4104/pcrj.2010.00053 20842323 PMC6602264

[21] IBGE/Diretoria de Pesquisas Coordenação de População e Indicadores Sociais. Gerência de Estudos e Análises da Dinâmica Demográfica. Projeção da população do Brasil por sexo e idade para o período 2000–2060. 2013 [Cited 01/03/2016]. Available from: http://www.ibge.gov.br/home/estatistica/populacao/projecao_da_populacao/2013/default.shtm

[22] Departamento Administrativo Nacional de Estatistica(DANE) Proyecciones de Poblacion 1985-2020. 2010 2010 [Cited 01/03/2016]. Available from: https://www.dane.gov.co/index.php/poblacion-y-demografia/proyecciones-de-poblacion

[23] Instituto Nacional de Estadisticas y Censo Estimaciones y proyecciones de la población total, por sexo y edad 1950-2050. [Cited 01/03/2016]. Available from: https://www.contraloria.gob.pa/inec/Publicaciones/Publicaciones.aspx?ID_SUBCATEGORIA = 10&ID_PUBLICACION = 474&ID_IDIOMA = 1&ID_CATEGORIA = 3

[24] Lee VJ, Chen MI, Yap J, Ong J, Lim WY, Lin RT, Barr I, Ong JB, Mak TM, Goh LG, et al. Comparability of different methods for estimating influenza infection rates over a single epidemic wave. Am J Epidemiol 2011; 174:468-78; PMID:21719743; http://dx.doi.org/10.1093/aje/kwr113 21719743 PMC3148265

[25] Jefferson T, Rivetti A, Di PC, Demicheli V, Ferroni E Vaccines for preventing influenza in healthy children. Cochrane Database Syst Rev 2012; 8:CD004879. 10.1002/14651858.CD004879.pub4 PMC6478137 22895945

[26] Jefferson T, Di PC, Rivetti A, Bawazeer GA, Al-Ansary LA, Ferroni E Vaccines for preventing influenza in healthy adults. Cochrane Database Syst Rev 2010:CD001269; PMID:20614424 20614424 10.1002/14651858.CD001269.pub4

[27] Jefferson T, Di PC, Al-Ansary LA, Ferroni E, Thorning S, Thomas RE Vaccines for preventing influenza in the elderly. Cochrane Database Syst Rev 2010:CD004876; PMID:20166072 20166072 10.1002/14651858.CD004876.pub3

[28] Ministério da Saúde SINAN. Influenza. Distribuição dos casos e óbitos por SRAG segundo Região/Unidade Federada de residência e vírus identificado. 2014 [Cited 10/05/2016]. Available from: http://portalsaude.saude.gov.br/index.php/situacao-epidemiologica-dados-influenza

[29] Paulo São Secretaria de Estado da Saúde de São Paulo. Centro de Vigilância Epidemiológica Prof. Alexandre Vranjac. Boletim Epidemiológico 2010-2013 2014. [Cited 14/09/2016]. Available from: http://www.saude.sp.gov.br/cve-centro-de-vigilancia-epidemiologica-prof.-alexandre-vranjac/

[30] World Health Organization FluNet. Laboratory confirmed data from the Global Influenza Surveillance and Response System (GISRS). 2014 [Cited 02/03/2016]. Available from: http://www.who.int/influenza/gisrs_laboratory/flunet/en/

[31] Carvalhanas TRMP, Paiva TM, Benega MA, Oliveira Santos KCO, Silva DBB, Ferreira PM, Paulino RS, Yu ALF, Rodriguez M, Liphaus B, et al. Influenza B circulation in Brazil and characterization of 75 B strains isolated from patients from São Paulostate, Brazil (2002-2013) [abstract]. 32nd Annual ESPID Meeting 2014; Dublin, Ireland European Society for Paediatric Infectious Diseases.

[32] Clements KM, Meier G, McGarry LJ, Pruttivarasin N, Misurski DA Cost-effectiveness analysis of universal influenza vaccination with quadrivalent inactivated vaccine in the United States. Hum Vaccin Immunother 2014; 10:1171-80; PMID:24609063; http://dx.doi.org/10.4161/hv.28221 24609063 PMC4896600

[33] Tricco AC, Chit A, Soobiah C, Hallett D, Meier G, Chen MH, Tashkandi M, Bauch CT, Loeb M Comparing influenza vaccine efficacy against mismatched and matched strains: a systematic review and meta-analysis. BMC Med 2013; 11:153; PMID:23800265; http://dx.doi.org/10.1186/1741-7015-11-153 23800265 PMC3706345

[34] DiazGranados CA, Denis M, Plotkin S Seasonal influenza vaccine efficacy and its determinants in children and non-elderly adults: a systematic review with meta-analyses of controlled trials. Vaccine 2012; 31:49-57; PMID:23142300; http://dx.doi.org/10.1016/j.vaccine.2012.10.084 23142300

[35] Bautista LE, Orostegui M, Vera LM, Prada GE, Orozco LC, Herran OF Prevalence and impact of cardiovascular risk factors in Bucaramanga, Colombia: results from the Countrywide Integrated Noncommunicable Disease Intervention Programme (CINDI/CARMEN) baseline survey. Eur J Cardiovasc Prev Rehabil 2006; 13:769-75; PMID:17001217; http://dx.doi.org/10.1097/01.hjr.0000219113.40662.dd 17001217

[36] World Health Organization WHO recommendations on the composition of influenza virus vaccines 2002-2012. 2015 [Cited 01/03/2016]. Available from: http://www.who.int/influenza/vaccines/virus/recommendations/en/

[37] Brasil. Ministério da Saúde Campanha nacional de Vacinação contra gripe 2014. Portal da Saúde. DATASUS. 2015 [Cited 10/05/2016]. Available from: http://pni.datasus.gov.br/consulta_influenza_14_selecao.asp

[38] Molinari NA, Ortega-Sanchez IR, Messonnier ML, Thompson WW, Wortley PM, Weintraub E, Bridges CB The annual impact of seasonal influenza in the US: measuring disease burden and costs. Vaccine 2007; 25:5086-96; PMID:17544181; http://dx.doi.org/10.1016/j.vaccine.2007.03.046 17544181

[39] Grupo Coomeva Información de bases de datos de cuentas medicas de Coomeva a nivel nacional, 2013.

[40] Panama. Ministerio de Salud Sistema Electrónico de Información de Salud (SEIS) 2010–2013. [Cited 25/01/2016]. Available from: http://www.minsa.gob.pa/programa/sistema-electronico-de-informacion-de-salud-seis

[41] Panama. Ministerio de Salud. Departamento de Epidemiología, Sección de Estadística de Vigilancia. Base de Datos/SISVIG. 2011-2013. [Unpublished raw data].

[42] Thompson WW, Shay DK, Weintraub E, Brammer L, Bridges CB, Cox NJ, Fukuda K Influenza-associated hospitalizations in the United States. JAMA 2004; 292:1333-40; PMID:15367555; http://dx.doi.org/10.1001/jama.292.11.1333 15367555

[43] Centers for Disease Control and Prevention (CDC) Estimates of deaths associated with seasonal influenza - United States, 1976-2007. MMWR Morb Mortal Wkly Rep. 2010 8 27; 59(33):1057-62; PMID:20798667 20798667

[44] Instituto Nacional de Estadistica y Censo (INEC) Defunciones en la Republica. 2007–2013 [Cited 25/01/2016]. Available from: https://www.contraloria.gob.pa/inec/Publicaciones/Publicaciones.aspx?ID_SUBCATEGORIA = 7&ID_PUBLICACION = 633&ID_IDIOMA = 1&ID_CATEGORIA = 3

[45] Porras-Ramirez A, Alvis-Guzman N, Rico-Mendoza A, Alvis-Estrada L, Castaneda-Orjuela CA, Velandia-Gonzalez MP, Hoz-Restrepo F [Cost effectiveness of influenza vaccination in children under 2 years old and elderly in Colombia]. Rev Salud Publica (Bogota ) 2009; 11:689-99; PMID:20339595; http://dx.doi.org/10.1590/S0124-00642009000500002 20339595

[46] Porras-Ramirez A, Mendoza AR, Montoya JM, Cotes K, Lopez JD, Herrera D, Rey G, de la Hoz F [Mortality associated with peak seasons of influenza virus circulation in Bogota, Colombia, 1997-2005]. Rev Panam Salud Publica 2009; 26:435-9; PMID:20107695; http://dx.doi.org/10.1590/S1020-49892009001100008 20107695

[47] IBGE Monthly employment survey - Economically active population. 2014 [Cited 15/09/2016]. Available from: http://www.ibge.gov.br/english/estatistica/indicadores/trabalhoerendimento/pme_nova/defaulttab_hist.shtm

[48] Colombian Central Bank Employment and unemployment rates - workforce percentage. 2014 [Cited 14/09/2016]. Available from: https://www.banrep.gov.co/en/node/24442

[49] Instituto Nacional de Estadistica y Censo (INEC) Encuesta de Mercado Laboral. 2014 [Cited 14/09/2016]. Available from: https://www.contraloria.gob.pa/inec/Publicaciones/Publicaciones.aspx?ID_SUBCATEGORIA = 38&ID_PUBLICACION = 636&ID_IDIOMA = 1&ID_CATEGORIA = 5

[50] Brasil. Ministério da Saúde Sistema de Gerenciamento da Tabela de Procedimentos, Medicamentos e OPM do SUS. DATASUS. 2014 [Cited 23/03/2016]. Available from: http://sigtap.datasus.gov.br/tabela-unificada/app/sec/inicio.jsp

[51] World Health Organization WHO-CHOICE. Health service delivery costs. 2008 [Cited 10/05/2016]. Available from: http://www.who.int/choice/cost-effectiveness/inputs/health_service/en/

[52] Chit A, Roiz J, Briquet B, Greenberg DP Expected cost effectiveness of high-dose trivalent influenza vaccine in US seniors. Vaccine 2015; 33:734-41; PMID:25444791; http://dx.doi.org/10.1016/j.vaccine.2014.10.079 25444791

[53] Organisation for Economic Co-operation and Development (OECD) Consultations with doctors. health at a glance 013: OECD Indicators. 2013 [Cited 01/06/2016]. Available from: http://dx.doi.org/10.1787/health_glance-2013-32-en

[54] Irving SA, Patel DC, Kieke BA, Donahue JG, Vandermause MF, Shay DK, Belongia EA Comparison of clinical features and outcomes of medically attended influenza A and influenza B in a defined population over four seasons: 2004–2005 through 2007–2008. Influenza Other Respir Viruses 2012; 6:37-43; PMID:21668663; http://dx.doi.org/10.1111/j.1750-2659.2011.00263.x 21668663 PMC4941556

[55] Falleiros Arlant LH, Bricks LF Influenza B burden in Latin America and potential benefits of the New Quadrivalent vaccines. J Pediatric Infect Dis Soc 2016; 5:1-2; PMID:26803330; http://dx.doi.org/10.1093/jpids/piv107 26803330

[56] Caini S, Huang QS, Ciblak MA, Kusznierz G, Owen R, Wangchuk S, Henriques CM, Njouom R, Fasce RA, Yu H, et al. Epidemiological and virological characteristics of influenza B: results of the Global Influenza B Study. Influenza Other Respir Viruses 2015; 9 Suppl 1:3-12; PMID:26256290; http://dx.doi.org/10.1111/irv.12319 26256290 PMC4549097

[57] World Health Organization WHO guide for standardization of economic evaluations of immunization programmes. 2008 [Cited 29/04/2016]. Available from: http://www.who.int/vaccines-documents/

